# The Pathology of Hysteria

**Published:** 1872-07-01

**Authors:** Norman Bridge


### THE PATHOLOGY OF HYSTERIA.

*A Paper read before the Chicago Medical Society, July* 15, 1872.

BY NORMAN BRIDGE, M.D.

Few diseases have been more carefully described, or, in symptomatic phenomena, are by the medical faculty better understood than hysteria. Yet in explaining the real nature of the diseased action, the pathology of the affection, there has been great difficulty, the causes and symptoms of the disease being often strangely confounded with each other, and with the way in which one leads to the other.

All are familiar with the phenomena of hysteria and all know about many of the causes, the conditions of bodily constitution and health, that lead to the strange manifestations. That certain peculiar mental states occur in many of the cases, and that in not a few there occurs a curious perversion of emotion and often as strange a lack of seeming sincerity and honesty, is all well known. It is less easy to see how any or all these conditions are related to such symptoms—how the latter are produced.

It seems to me that much of the difficulty of the question comes from our constant habit of looking for some lesion of an organ, some one poisonous agency or morbific influence as the cause of disease. It is the nature of our minds to do this. We find one poison to produce small-pox, or measles, or malarial fever; one agency to bring on pneumonia or rheumatism, and are satisfied. We examine the organs and influences of mind and body for some single thing as the cause of hysteria. We fail to see how any one of our supposed causes could possibly lead to all the phenomena of the disease, and so we are not satisfied.

There is not found any one lesion of body to be a constant attendant on hysteria; disease of the uterus has been thought to be such, but so far is this from the truth, that persons without the uterus have the disease; probably in a majority of cases of hysteria no lesion whatever is discoverable.

So far as our insight can reach, the affection is, as near as any disease may be, a functional one; and its functional manifestations are of the nervous system, and of that part of the nervous system closely connected with the part where resides—to use a common phraseology—the mind. Indeed, in more than half the cases of hysteria, certain phases of mental manifestation are involved as elements of the trouble. What is the sly plot, the intrigue of the patient to arouse the sympathy of others in her case, but an operation of mind? And the hysterical convulsions into which a patient falls when bystanders are discussing her afflictions; could there be a better example of certain trains of thought on the body?

Nor is the cerebro-spinal the only part of the nervous system that is involved. The sympathetic, in all its influence over the circulation in different parts and over the operations of the visceral and glandular organs, shows that it too becomes included in the general diseased action.

The nervous system in its physiology, and more in its pathology, is a labyrinth. Yet I fail to see how the pathology of hysteria can be understood, save by a study of its phenomena of disease, from the standpoint of the physiology of the mind and higher nervous functions.

The phenomena of the disease are to a great extent phenomena of the mind, and such as may arise from the effect of the mental nature on the body, and we ought to study them as we would any other psychical phenomena—as we would insanity. Until insanity began to be studied from this standpoint the insane were locked up and treated like wild beasts, like murderers and those possessed of devils. Hysteria has been regarded as a condition of moral obliquity, as a synonym of deception and often of ugliness; physicians avoid telling their patients they have the disease, lest the statement might be regarded as an insult, as an accusation of lying would be. When the physiology of all mental acts comes to be more understood this humiliating condition of things may, let us hope, be bettered.

The operations of mind in all its phases—with all its faculties, supposed to be different things—more truly different modes of action of one great organ — its connection with organs of the body, its influence on them and their influence on it; all these actions, interactions, and relations, make of course a study of singular complexity. Yet only by going into the detail of these things can we arrive at the correct nature of any disease in which mental operations play a chief part—of a disease like hysteria.

Nearly all actions, and many thoughts, to which human beings are impelled, have for their directing influence some feeling of pleasure or pain to the individual doing them. This is the essence of the first law of our natures—self-feeling, love of the things that will profit and please, and dislike of the things that may in any way cause pain.

This feeling gives color to thought, it influences and moulds thought and reasoning, and the two together make what we call *emotion*. The emotional nature which we are told has so much to do with hysteria is simply that capacity or faculty by which the operations of reasoning and abstract thought are influenced by this native self-feeling. One in whom this control is great is said to have a powerful emotional nature.

It is that faculty out of which directly or indirectly come the sympathies, the affections and attachments, the envy, hate and jealousy. Women have it more than men, but all people have it. It is distinct and has a different seat in the brain from reason or ideation.

A man can reason as well on mathematics, law, or medicine, without the emotions; in his reasoning they are not required. His reason deals with abstract things; his emotions deal with himself. Reasoning is an endowment by which we are able to comprehend the relation of things in such fashion that we are called superior beings; that which is the main-spring of the emotions is the endowment by which we are made to care for self, to have associations and society; out of it grows most of the amenities of life, and by it are developed elements of our better nature.

The emotional processes have their seat, according to authors, in the sensori-motor centers—the optici tholami, corporæ striatæ, and tuberculæ quadrigeminæ, the ganglionic nuclei of the nerves of the special senses, and possibly the cerebellum, the ideational function being carried on by the cerebral hemispheres.

This mass of brain substance—the superior lobes—is unconnected with the body by any nerves save what pass through the sensori-motor centers; it is hence placed beyond the reach of impressions or sensations from without, and is unable to send any impressions to the body except as they all pass through the last named centers. These centers in the base of the brain then, receive all the nervous impressions that come from the body; in them, by the relaxation of these impressions, most of the impulses—not the ideas—originate. From these centers the impressions that are made upon them pass up to the ideational centers, and through them the volitions of these upper centers are made into actions through the bodily organs. If the impressions made on the sensori-motor centers are brief or slight, they give use to sensation and reflex action directly, without affecting the center of ideation, while if they are severe or long continued, they are transmitted upward to the centers of reason. The sensori-motor are the only centers which affect directly the bodily organs, and it is they that are, of all the brain centers, affected by them; it is to them the organs in some way make known their needs and their complaints.

This sympathy between the mind centers and the organs must be kept constantly in view; as a force in our physiology it is almost all-controlling. The condition of every organ of the body influences the state of mind; their various states lead to nearly all the moods. Irritation of the lung substance, as in consumption, causes a buoyant mood; that of the liver, one of despondency and gloom; a patient with heart disease is kept in horrid suspense of mind, while any irritation of the sexual organs, as is well known, influences the mood of mind more than that of any other part.

It is not unlikely that nearly every morbid mood into which we fall, if not directly due to some external cause, may be explained by some state of bodily organs. And if irritation of an organ for a short time may evidence a mood, it may, long continued, make that mood a fixed mental state, a characteristic of mind, a part of individual character.

In this way men, from irritation of stomach, become habitually morose, so that on a cure of the dyspepsia the mental state does not entirely recover; men are made chronically melancholy by some irritation of liver or heart, and insane about self-abuse of the sexual organs.

Not only do bodily conditions determine mental states, but every unusual mood of mind directly reflects on the organs of the body and tends to induce that condition which, itself otherwise induced, would cause the mood. The familiar effects of mental states on the action of glands, of digestive organs, of the heart, and on the sexual system, are only a few examples illustrating this principle.

If again a certain frame of mind, momentarily induced, can so affect bodily organs, the condition long continued or made habitual, must induce permanent changes in the health and condition of such organs.

Mental states, besides largely dertermining the functional activity of visceral and glandular organs, induce all sorts of muscular action and of sensation, evincing the sympathy between mind and the animal nervous system. The range of this influence is very wide and varied. A physician in this city has been subject for many months to attacks of gastralgia or of sciatica whenever he engages in difficult study. A gentleman connected with the press has, for several years, whenever engaged in perplexing brain-work, felt a pain in the back side of his head, on the right side of the mesial line. People subject to all degrees of chorea are frequently thrown into the worst muscular action by mental embarrasment. Dr. H. Jones says, that "sudden fright causes epilepsy, and next to this fits of passion, distress of mind, and venereal excess," produce it. I am informed of an intelligent woman, now residing in this city, who, on becoming frightened or receiving any profound mental impression, has a severe pain the whole length of the spine, and this is the only symptom she has.

With these facts closely in mind, if we could gain fully one other principle of physiology, it seems to me we might get some insight into the correct nature of the production of hysteria.

That principle is this, that every faculty of mind—of nerve center—*grows by exercise.* This is true of memory, of reasoning, of ability to do in any one direction ; it is because of this fact that everything is learned, that we acquire the ability to do certain things, as it were, by intuition. The intuition is only a state of nerve-culture, inherited or gained by habit, by which certain acts are performed more easily than others.

Every tendency of mind that is abnormal, if persisted in, becomes the settled habit of the individual; it becomes with him automatic; it is a part of himself; it has come to be an instinct. This seems to be more true of the emotional than any other manifestation of mind.

Now, at any time, and in any organism, emotion has a strong influence on bodily functions. If any phase of emotion is persistently indulged in, repeatedly exercised, or stimulated, its power over the body and being will grow to be almost supreme. This occurs from the close sympathetic connection between the general nervous system and the sensori-motor centers ; the phenomena are morbid in character as well as time and place, and the emotion acts as any other abnormal stimulation might to produce them. So powerful is this, that in one in whom the emotional nature has become, by constant stimulating and pampering, or by any means overgrown, any slight disturbance causes the muscles of the body to be thrown into violent contortions, the sensation of choking to be felt in the throat, or violent weeping or laughter to be manifested. We call it hysteria, but it does not differ in nature from many phenomena in persons not hysterical.

But this case supposed is a form of hysteria in which evidently only the emotional centers — the sensori-motor — are involved. Such patients will show no signs that the reasoning powers—the ideational centers—are at all disordered. They will be conscious of what they have been doing, will be free to talk and reason about it, and not unlikely will reproach themselves for being silly and not having more self-control. Their logic, whatever else happens, does not get faulty.

The more common form of hysteria is that in which the erethism of the emotional centers has affected those of ideation—the same sequence of events absorbed in most regularly progressive insanities—and the other symptoms are joined with wrong mental action, distorted conception of things, and the patient comes to have a fixed delusion. This often makes her to indulge in all shades of deception and intrigue, even to the subjecting herself to severe suffering, to keep up the belief in her ills and to arouse for herself sympathy.

This patient is insane; and I fail to comprehend how her case differs, in the nature of the elements involved and in their order, from that of any true ideational insanity of a mild type. In insanity the first step is disorder of the sensori-motor centers, giving hallucination to erratic conduct; then this disorder is extended on upward to the superior centers, the seat of ideation, and there is an outright delusion; the man's reasoning is deceptive and wrong. How closely does this agree with the description of the steps in the progress of hysteria.

Patients with the latter form of hysteria will never acknowledge their erratic conduct, will never talk about it with an open frankness; if spoken to in reference to it, they will evade the subject, or break out in laughter or crying.

One of the most positive proofs that ideational insanity, in any case, still exists, is that the patient remains utterly oblivious of the fact that delusion has existed; a clear proof of ideational hysteria is the persistent evasion of the subject by the patient.

Now, let the symptoms and conduct due to this mental and nervous state—the hysterical symptoms—be often repeated, and the cerebro-spinal axis becomes so habituated to them, that it will, on slight disturbance of any sort, cause them to be reproduced. It becomes a habit of the nerve centers, and can no more be prevented than the lower animals can avoid doing what they are by instinct impelled to do.

So it happens, that in these confirmed hysterics, the fits continue to recur long after the patient has honestly tried to stop them. They come on without her consciousness even; they are her automatonism.

In the way of its production and in its *modus-operandi* a case of this description cannot differ very far from a case of confirmed epilepsy, the first fits of which were induced by act of will; several such cases being on record. It is even more like a case of confirmed epilepsy, the first paroxysms of which were caused by reflex irritation of some sort, in a weak and nervous system; which case, by the way, is exceeding common.

In the worst cases of hysteria then we have, to begin with, a perfectly natural emotion. From some disease of an organ, some weakness of organization it may be, this emotion is too much indulged in; then comes out of it the natural love of being observed, the desire for sympathy and pity. The more the mind dwells on the pity of others, the more rapidly grows the feeling, and she comes to care more for the sympathy others have for her than she does to recover from any infirmity. Her chief enjoyment comes to be in the consideration and attention of others and the thoughts about herself; the thought comes to be painful that a time must come when, by her recovery or otherwise, this sympathy will be withdrawn. It is only a short step from this condition, only a slight change in the state of mind, to one in which she will, by systematic planning, keep up the interest and wonder in her case, in which she will deceive others and injure herself to accomplish this purpose.

At first thought such a gradual change from a normal mental state to one of such perversity, seems absurd and wholly unaccountable, except on the theory of thorough depravity. But when the strong love of self, of appearance, of popularity, of being thought remarkable or peculiar, which are such constant parts of all our natures, are considered, it is so far from strange that a little stimulation or overgrowth of the emotions should lead to all these, the symptoms of hysteria, that the greater wonder is we do not see ten times as much of the disease as we do.

But, however caused, the nervous symptoms of hysteria—the choking, crying, laughing, paralysis, spasmodic contraction of muscles, and diseased sensations—must be regarded as hardly possible to occur in any but a nervous system in some way weakened, by overwork or other cause of debility; with such debility it is always more easy for any similar symptoms to be produced.

With all debility of nervous and muscular tissues, their irritability is increased; and the more easily are sensations and motions induced. They occur on slighter stimulation, and are made more active on the ordinary normal stimulation. This is a law of the system, of the physiology, which is pathology; it further seems to be a law of the system that, as this debility comes on, and this irritability increases, the tendency grows for the various functions to be unnatural in character, as are the spasms and morbid sensations. Yet these erratic phenomena, the spasms of all sorts, the pains and abnormalities of sensation, are little more than what might be produced by simple increase or decrease in the normal function.

We have a multitude of examples of this erratic action in various diseases besides hysteria, and in many others besides this affection the mental state acts as a stimulant or irritant to the sensitive motor centers to produce the phenomena.

Epilepsy is one of them, directly and clearly produced by mental action; by fright, anger, and other depressing passions, as well as outright intention to produce it. Chorea is due very frequently to fright and terror in children, and is vastly aggravated by mental worry and embarrassment; it occurs, moreover, chiefly in children, stamping it as a disease of delicate nervous systems.

Indeed chorea, epilepsy, and hysteria seem to be convertible conditions, insomuch that they frequently take the place of, and are substituted by, one another. The jerking in torticollis and even asthma are aggravated and occasionally induced by emotion.

But we ought to expect that fatigue alone of the nervous system would, in certain sensitive natures, give rise to many of the symptoms of hysteria. It would require a peculiar organization doubtless, but we have analogue in the case of convulsions of children from debility, in which the text-books caution us against treating the cases for cerebral inflammation; also in the case of violent struggling of men and animals on losing suddenly a large quantity of blood—in which there is debility suddenly induced of the nervous system, but no outright lesion of it.

It is no more remarkable that hysteria should, in its symptoms, be produced likewise by debility, and it is the clinical fact that cases of this character frequently occur; cases in which there is no abnormality of emotions whatsoever.

Thus we have three forms of hysteria:—(1.) That due to overgrown emotions acting on a weakly nervous organism, but without will or ideational function entering into it as elements. (2.) That in which, in addition to these conditions, the ideational powers are involved—purposeless deception and delusion. (3.) That in which the only pathological condition consists in an overtiring of an already sensitive, nervous system.

Probably the ultimate nature of many of the symptoms of hysteria no man, in the present state of pathological science, can fully explain; but what I am anxious we should keep in view in this discussion is, that the functional symptoms differ in nature very little from those of many other diseases, such as epilepsy, chorea, and anæmia; that they are not the outgrowth of any new force operating in the system, of anything introduced specially into it, but are due to the forces and powers that are natural, and these may occur in any person.

We may invoke congestion and anæmia of nerve centers and their paresis to explain these functional symptoms; we may say that reflex irritation causes them, or attribute them to any other of the palpable causes which even lead to such symptoms, and we will probably be doing a perfectly warrantable thing, for all such agencies doubtless do operate at times in producing the phenomena. But we must keep in mind that exactly the same causes induce the symptoms here as in other diseased conditions, and when we get at the explanation of them elsewhere, we will have the science of them in hysteria. Above all we must remember that habitual exercise of any nervous center, faculty, or element, tends to be repeated and to have increased power in this direction, and that in this way abnormalities may easily grow up through the emotional condition, giving us all the phenomena of hysteria. This fact, that the abnormal functions of sensation and motion may be induced by the character of development of faculties of mind—really the automatonism of those nerve centers most closely connected with the bodily functions—seems to me to be the most distinctive feature of the pathology of the disease under consideration.

The foregoing view leaves untouched some questions regarding this disease which deserve consideration. Among them is the question, first, of what part disease of the uterus plays as a cause of the malady ; and, second, why women have the disease more than men.

There are large numbers of women who, with every form of disease of the uterus, are entirely free from hysteria, and there are but very few who seem to be thrown into the hysterical state solely by disease of this organ. These facts are well substantiated, and seem to warrant us in assuming that disease of the uterus does not act in any marked manner as a cause of hysteria.

But there is a way in which we conceive the condition of the uterus has much to do with this affection. Hysteria is pre-eminently a disease of the emotions, which perhaps, stated differently, is to say, that hysteria, in a majority of cases, is some unsatisfied like or dislike. I am not sure but this division of hysteria would be properly defined as a disease of the nervous system due to an unsatisfied longing for some stimulation—the stimulus, the longing, or both, being morbid.

The emotions are pre-eminently the outgrowth of the states of the sexual organs, in both sexes. Not that emotion cannot exist save as determined by these organs, but they are more vital and absolute in the control their condition has over the emotions, than almost all other organs put together. The change in the life of the eunuch by his castration is chiefly in his emotions. Men born with testicles atrophied, or in whom this change has spontaneously occurred afterwards, and women without ovaries, illustrate equally this principle. But the various states of quiescence and stimulation of these organs that we see every day in naturally formed people, prove this law better than any condition of monstrosity.

If emotion hence can be induced at all, or directed, it is most easily done by some state of the sexual system. In proportion to the early development or the active nutrition of the uterine system, in proportion to the irritation of this system, we may fairly conclude would be the vigor of the emotions. This is what observation shows to be true. Hysteria does not most often occur in women with diseased uteri ; but in those whose sexual systems, by the pampering of society life, by a self-life in lascivious thoughts, or by a very rapidly growing physical frame, are highly developed and sensitive; in those in whom this system is active in its control of the functions.

The male sex illustrates the same principle. When there is real organic disease of the sexual organs, as inflammation or ulceration, there is seldom much change in the emotional state; but let these organs be over-stimulated, as in boys addicted to abuse of self, or in persons who indulge in venereal excesses, and see how quickly the peevishness, egotism, jealousy, and unaccountable temper, pronounce a state identical with hysteria. The trouble is too much excitement of the sexual system. Take again the case of an elderly man who, after years of regular and legitimate sexual indulgence, is suddenly deprived of it, and the restraints of society compel his continence, and note how soon his emotional nature discloses the fact; he indulges in sickly sentiment, in writing poetry, he thrusts himself into female society, and does a dozen other things he would before have believed himself incapable of.

Let the emotional nature be aroused by any other cause and there can be no question it would and does act directly upon the nutrition of the uterine system to stimulate it and make it sensitive. The two states are accompaniments of each other. So it must happen that, with the increased emotional activity, there would be a congested, overstimulated, sensitive condition of the uterine system.

Such conditions are always most conducive to the development of diseases of the uterus; they are so written down in all the textbooks on gynæcology, and beyond question correctly so written.

The uterine disease and the hysteria then are, as a rule, like results of the one cause, and not respectively cause and effect, and I believe this is the only and the true relation between the two conditions.

This view too explains the fact that women have more hysteria than men. The uterine function in woman's physiology plays a more important part than any function, than almost all the functions in the body of man, except only those functions called vital; all of which is sufficient reason why she might be expected to have more emotion than man. And perhaps this is the only way in which the evident plan of her creation was to be carried out, by which to fit her for the highest office human beings fill—that of motherhood—it was evidently meant she should have less coldness and more emotion and tenderness than man.

Her weaker physical frame and her liability to frequent physiological drains on her vital powers, are sufficient causes for her giving way to the emotions more readily and having less nervous self-control than man. And this explains the increased hysterical tendency.

