# Errata

**Published:** 1872-08-15

**Authors:** N. Bridge

**Affiliations:** 267 W. Monroe St., Chicago


Errata.—By a mistake the proofs of Dr. Bridge's article were not sent to him for correction; hence the following letter:

*Editors of the Examiner:* In the paper published in the Examiner of July 1st ult., entitled "The Pathology of Hysteria," there are fifteen typographical errors, most of them so patent and glaring, that should they go uncorrected it were better for both author and publishers that the article had never seen the light. In the second column of page 193, the sentence "could there be a better example of certain trains of thought," etc., should have after the word "example" the words "of the effect." In the second column of the next page, "by the *relaxation*," should read, "by the *reflex action*;" and a few lines below, "they give *use* to sensations," should be, "they give *rise*," etc. In the first column of page 195 the print says, "may *evidence* a mood,"—"may *induce*," it should be. In the next paragraph, "insane about self-abuse," in the manuscript read "insane about self *by* abuse," etc. In the next column, "*nerve culture*," should be "*nerve-center*." The word following the words "sequence of events," on the next page, should be "*observed*," not "*absorbed*." Below, on the same page, the phrase "giving hallucination *to* erratic conduct," should have *and* instead of *to*. In the second column of this page the sense is distorted by the type saying "then comes out of it the natural love of being observed," etc.; "*and* the natural love of being observed," it was written by the author. On the next page is a phrase the reader of the paper intended as a very fine thing—"the physiology which is pathology"—yet see how utterly it is spoiled by the printer putting after the word *physiology* a comma! Instead of "*these*," the last word of the first line of page 198, should be "*that they*;" and six lines below, the word "*even*" should be "*ever*." Very respectfully,

N. Bridge.

267 W. Monroe St., Chicago.

